# From spore to gametophyte: Investigating morphological dynamics of hyaline cells and corresponding gene expression patterns in *Sphagnum*

**DOI:** 10.1186/s12870-025-06770-w

**Published:** 2025-06-07

**Authors:** Xianlin Guo, Fumin Xie, Chaojie Wang, Kai Fang, Dan Xue, Liangfeng Liu, Xinwei Liu, Xiaohan Yang, Yuchen Huang, Tianyao Han, Huai Chen

**Affiliations:** 1 Mountain Ecological Restoration and Biodiversity Conservation Key Laboratory of Sichuan Province, Chengdu Institute of Biology, Chinese Academy of Sciences, No. 23, Qunxian South Street, Tianfu New Area, Chengdu, 610213 China; 2 College of Agriculture and Biological Sciences, Dali University, Dali, 671003 China

**Keywords:** Hyaline cell, Morphology, Phylogeny, *Sphagnum*, Transcriptome

## Abstract

**Background:**

Peatlands play a vital role in mitigating climate change and maintaining global ecological balance. At the core of these ecosystems are *Sphagnum* mosses, which rely on their hyaline cells—specialized structures with exceptional water retention capacity—to stabilize wetland hydrology and support long-term ecosystem function. Despite their ecological importance, the molecular mechanisms underlying the development of these unique water retention cells remain poorly understood. This study focuses on *S. capillifolium*, examining morphological traits and gene expression dynamics across four developmental stages to uncover how gene regulation contributes to hyaline cell formation.

**Results:**

During the transition from spore germination to mature gametophytes, significant morphological changes occur in the cell walls of hyaline cells, particularly their increased volume, which distinguishes them from chlorophyllous cells. Transcriptomic analyses revealed marked changes in gene expression related to the cell wall, apoplast, and extracellular regions during hyaline cell formation. Notably, genes associated with cell wall remodeling, such as *EXO70*, *PME*, and *XTH* genes, were significantly involved. Phylogenetic analysis uncovered evolutionary divergence in these genes, highlighting the unique evolutionary position of *Sphagnum* compared to vascular plants, forming an independent branch. Further protein structure analysis revealed distinct differences in the ligand-binding sites and hydrogen bond formation of EXO70, PME, and XTH proteins compared to vascular plants, which may account for the functional changes observed.

**Conclusion:**

This study reveals the gene expression patterns underlying hyaline cell development in *Sphagnum capillifolium*, with a focus on key genes involved in cell wall remodeling. The findings highlight significant evolutionary differences between *Sphagnum* and vascular plants, particularly regarding the unique functional and structural characteristics of *EXO70*, *PME*, and *XTH* genes. These insights provide a new perspective on the molecular mechanisms behind hyaline cell formation in *Sphagnum*, further enhancing our understanding of its role in regulating wetland hydrological environments.

**Supplementary Information:**

The online version contains supplementary material available at 10.1186/s12870-025-06770-w.

## Introduction

*Sphagnum* are vital components of wetland ecosystems, playing a key role in the formation and preservation of peatlands, which act as significant carbon sinks in the global carbon cycle [1–3]. Their remarkable water retention and carbon sequestration abilities are fundamental to the ecological stability of peatlands [4–7]. These mosses achieve this through specialized leaf structures and hyaline cells, which optimize water retention [5, 8–10]. Given the critical role of peatlands in mitigating climate change, understanding the molecular mechanisms behind *Sphagnum'*s water retention capacity is essential for preserving the hydrological integrity of these ecosystems.

*Sphagnum* undergoes several key developmental stages, including spore germination, protonema formation, and gametophyte maturation [11, 12]. The developmental progression from protonema to thalloid structures, accompanied by the differentiation of hyaline cells alongside photosynthetic chlorophylous cells, serves critical functions in water retention [13–15]. The formation of hyaline cells is influenced by multiple biological processes such as cell expansion, secondary wall thickening, and programmed cell death [16]. These processes are tightly regulated by water pores, which promote dynamic changes in the structure of the cell wall and secondary wall deposition, ultimately leading to the formation of large, rigid cells optimized for water storage. Despite the absence of vascular tissue, hyaline cells enable *Sphagnum* to adapt to waterlogged and nutrient-poor environments, highlighting their essential role in the species' ecological success [17–19]. Additionally, hyaline cells provide microhabitats for microbial communities, which are critical for ecosystem processes like nutrient cycling and carbon sequestration [4, 20].

Recent studies have identified several key genes involved in regulating cell wall remodeling and interactions with the extracellular matrix, such as *PME* (pectin methylesterase) and *XTH* (xyloglucan endotransglycosylase). These identified genes exert critical functions in mediating cell wall integrity dynamics and remodeling processes, while being indispensable for the structural assembly and functional maintenance of the cell wall [21–24]. Furthermore, the EXO70 subunit of the exocyst complex is involved in processes like cell elongation, polarization, and exocytosis, contributing to the structural and functional integrity of the cells [25–27]. However, while these findings have advanced our understanding of cell differentiation and cell wall remodeling, the complete molecular mechanisms driving the formation of these specialized cells remain unclear.

This study investigates the morphological and molecular processes involved in the development of *Sphagnum capillifolium*, covering four distinct stages: filamentous protonema, thalloid protonema, young gametophyte, and mature gametophyte. By examining gene expression patterns and structural variation associated with hyaline cell differentiation, this study aims to explore the molecular mechanisms that underpin the formation of hyaline cells, which are crucial for *Sphagnum*'s exceptional water retention in peatland ecosystems.

## Results

### Morphological development from spore to gametophyte

**Fig. 1:**
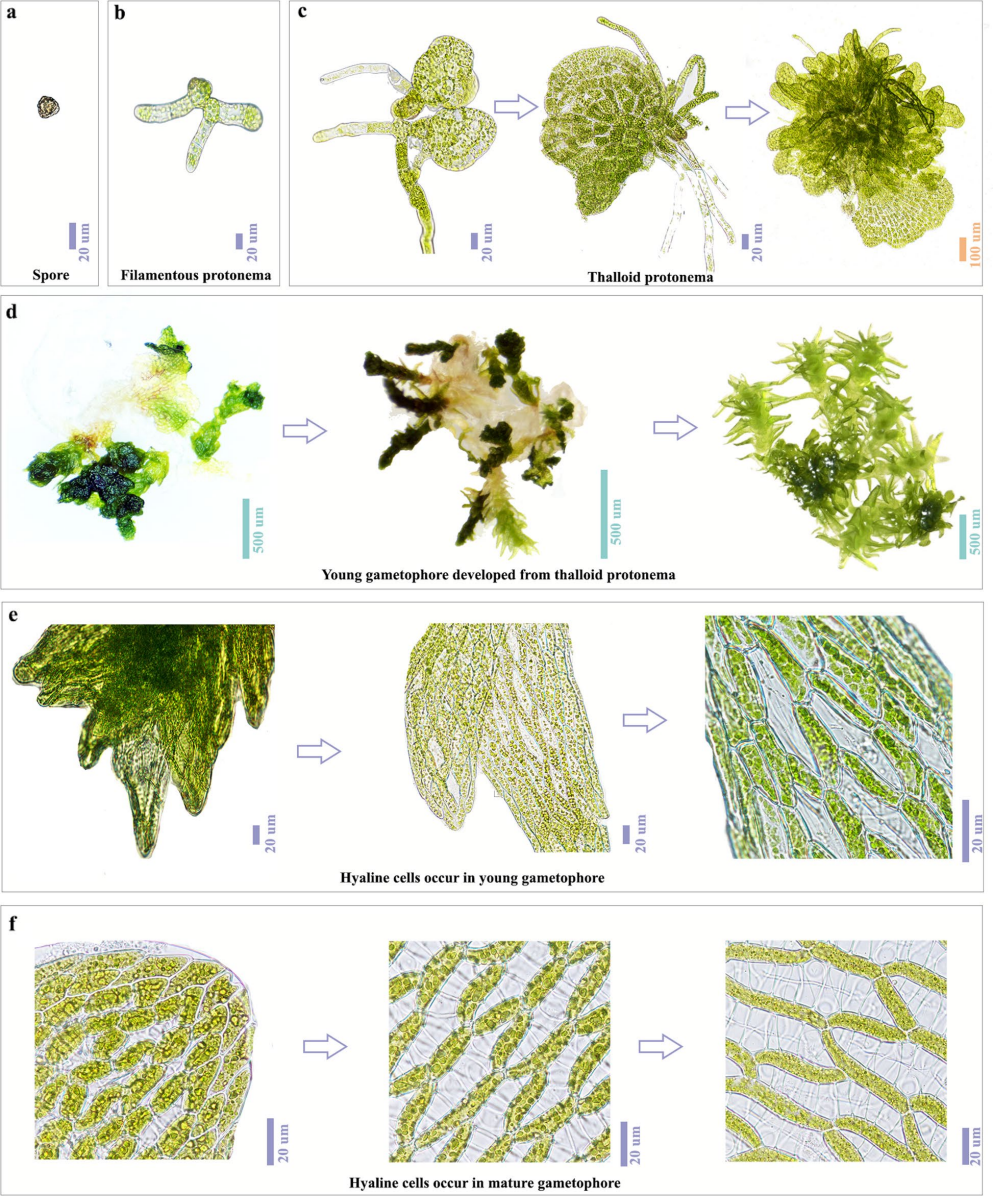
Morphological development of *Sphagnum capillifolium* leaves and cells from spore germination to the mature gametophyte. **a** Spores. **b** Cell morphology during the filamentous protonema stage. **c** Thalloid protonema stage, showing a gametophyte structure, primarily for anchorage, with multilayered thalloid structures on the right. **d** Transition from young to mature gametophyte. From left to right, the development of gametophyte-like structures and leaf expansion are depicted. **e** Cellular morphology during leaf expansion on the gametophyte structure. Transition from uniform cell types to the preliminary formation of hyaline cells is observed, with evident chloroplast degradation and cell differentiation. **f** Cellular morphology in the leaves of mature gametophytes. The leaf apex shows differentiated cells (left), the mid-leaf region exhibits enlarged hyaline cells and secondary wall growth (center), and mature hyaline cells display pores and secondary wall features (right)

The morphological development of *Sphagnum capillifolium* progresses through distinct stages: spore germination, filamentous protonema, thalloid protonema, young gametophyte, and mature gametophyte (Fig. 1). After spore germination, the organism forms a filamentous protonema that grows horizontally (Fig. 1a, 1b). As it develops, the protonema expands at the apex, forming a thalloid protonema. This plate-like structure grows, with root-like segments persisting throughout development (Fig. 1c). During the mid-stage, the thalloid protonema remains a single layer, but it later transforms into a multi-layered structure. Further differentiation on the protonema surface marks the transition to an upright, gametophyte structure with deep green leaves, distinct from the senescent protonema beneath (Fig. 1d). The gametophyte then develops a branched architecture, with ovate to lanceolate leaves that are concave at the apex and toothed along the edges.

At the cellular level, minimal differentiation occurs during the early stages, with cells containing chloroplasts for photosynthesis (Fig. 1b, 1c). As gametophyte development progresses, cellular differentiation becomes more evident. Initially, leaves show little specialization, but as development continues, some cells lose chloroplasts and form hyaline cells (Fig. 1e). These hyaline cells enlarge, develop spiral thickenings, and form water-conducting pores. This differentiation becomes more pronounced in mature gametophytic branches, where asymmetric cell division mainly occurs at the leaf apices, with spiral thickening concentrated in the mid-leaf region, and enlarged hyaline cells most prominent at the leaf base (Fig. 1f).

### Clustering of differentially expressed genes across developmental stages

**Fig. 2:**
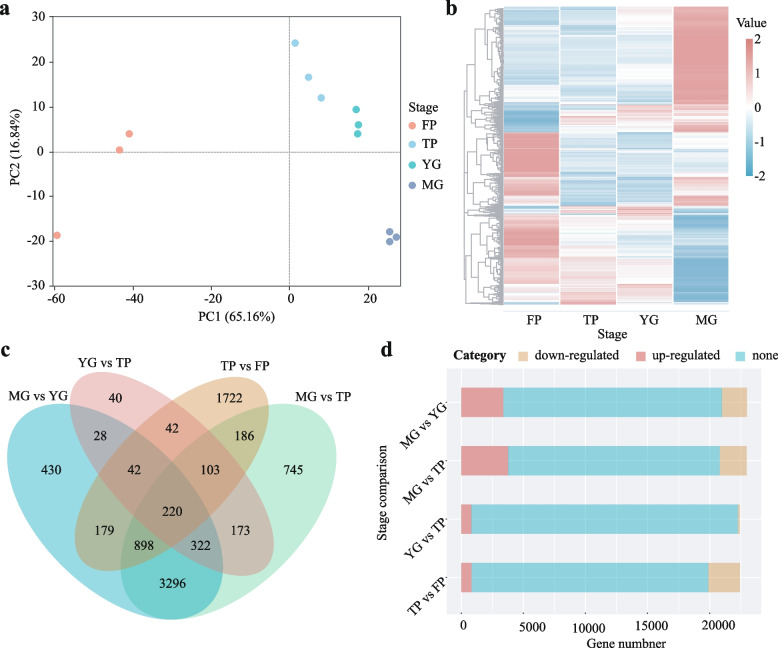
Transcriptomic analysis across four developmental stages of *Sphagnum capillifolium*: filamentous protonema (FP), thalloid protonema (TP), young gametophyte (YG), and mature gametophyte (MG). **a** Principal component analysis (PCA) showing distinct gene expression profiles for each stage, with strong correlations among biological replicates. **b** Gene expression pattern analysis reveals significant differences between FP and MG, while TP and YG show greater similarity, with TP's profile closer to FP. **c** Differentially expressed gene (DEG) analysis across four conditions (TP vs FP, YG vs TP, MG vs TP, MG vs YG). **d** Total differential genes, upregulated genes, and downregulated genes among four conditions (TP vs FP, YG vs TP, MG vs TP, MG vs YG)

Each developmental stage was sampled with three biological replicates, resulting in a total of 12 transcriptomic datasets (Table S1). Principal component analysis (PCA) revealed clear differences in gene expression across the four stages, with high correlation among replicates within each stage (Fig. 2a). Expression pattern analysis showed the most significant difference between filamentous protonema (FP) and mature gametophyte (MG), while thalloid protonema (TP) and young gametophyte (YG) were more similar, with TP closer to FP in expression profile (Fig. 2b).

Differential gene expression analysis identified the largest number of unique differentially expressed genes in the TP vs FP comparison (1,722 genes), while the TP vs YG comparison had the fewest (40 genes) (Fig. 2c). Across the four comparisons (TP vs FP, YG vs TP, MG vs TP, and MG vs YG), the number of differentially expressed genes ranged from 970 (YG vs TP) to 5,943 (MG vs TP) (Fig. 2d). MG vs TP showed the most upregulated genes (3,774), followed by TP vs FP (1,112) and MG vs YG (3,404). The number of downregulated genes was lower overall, with MG vs TP showing the fewest (143), and the others ranging from 1,024 to 2,169.

### Enrichment and annotation of differential genes across developmental stages

**Fig. 3:**
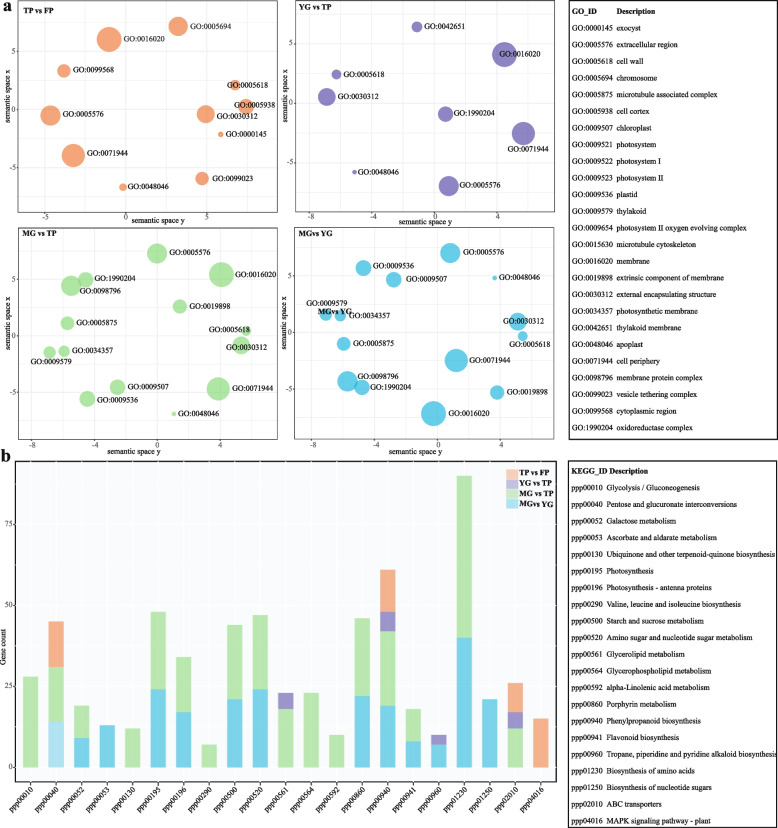
GO and KEGG pathway analysis of differentially expressed genes across *Sphagnum capillifolium* developmental stages. **a** GO analysis shows common enrichments in the apoplast, cell wall, and extracellular regions. **b** KEGG analysis reveals distinct pathways for each comparison

Gene Ontology (GO) analysis of differentially expressed genes across developmental stages revealed both common and stage-specific enrichments (Fig. 3a). All four comparisons showed enrichment in the apoplast (GO:0048046), cell periphery (GO:0071944), cell wall (GO:0005618), extracellular region (GO:0005576), and external encapsulating structure (GO:0030312), with these terms exclusively enriched in the TP vs FP comparison. The YG vs TP group additionally showed enrichment in membrane-related terms, including intrinsic (GO:0031224) and integral components of membrane (GO:0016020), with GO:0031224 uniquely enriched in this comparison. In contrast, MG vs TP and MG vs YG were enriched in photosynthesis-related GO terms, such as thylakoid (GO:0034357, GO:0009579, GO:0042651), photosystem components (GO:0009521, GO:0009522, GO:0009523, GO:0009654), extrinsic component of membrane (GO:0019898), oxidoreductase complex (GO:0009536), chloroplast (GO:0009507), and plastid (GO:0009536), distinguishing these stages from the others.

KEGG pathway enrichment analysis also revealed distinct profiles across stages (Fig. 3b). The TP vs FP comparison showed specific enrichment in the plant MAPK signaling pathway (ppp0416). In the MG vs TP comparison, enriched pathways included glycolysis/gluconeogenesis (ppp00010), ubiquinone and terpenoid-quinone biosynthesis (ppp00130), valine, leucine and isoleucine biosynthesis (ppp00290), and alpha-linolenic acid metabolism (ppp00592). The YG vs TP comparison showed enrichment in glycerolipid metabolism (ppp00561), phenylpropanoid biosynthesis (ppp00940), tropane, piperidine and pyridine alkaloid biosynthesis (ppp00960), and ABC transporters (ppp02010). In the MG vs YG comparison, unique enrichment was observed in ascorbate and aldarate metabolism (ppp00053) and biosynthesis of nucleotide sugars (ppp01250). Notably, phenylpropanoid biosynthesis (ppp00940) appeared in all comparisons, highlighting its key role in metabolic regulation across stages.

### Phylogeny and stage-specific expression of *EXO70*,* PME*, and* XTH* gene families

**Fig. 4:**
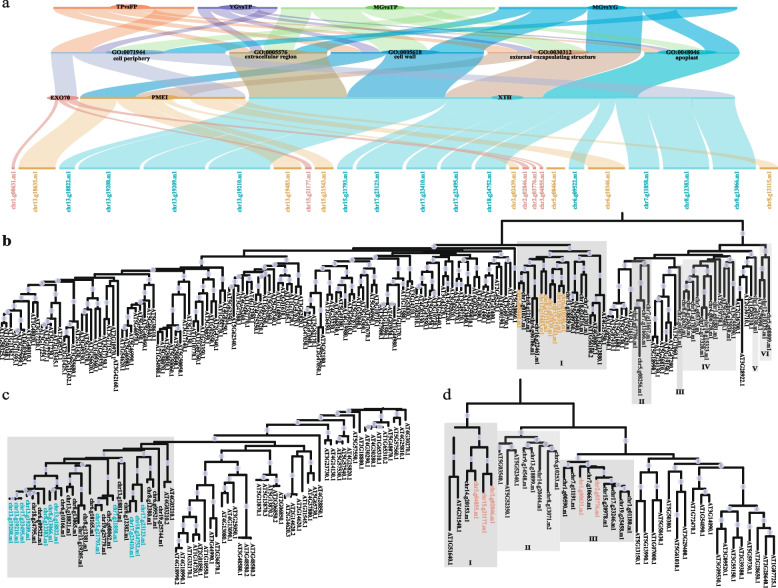
Phylogenetic and functional analysis of genes in *Sphagnum capillifolium* related to apoplast, cell periphery, and cell wall processes. **a** GO enrichment analysis of genes involved in the apoplast, cell periphery, cell wall, extracellular region, and external encapsulating structure. Genes from the *EXO*, *PME*, and *XTH* families are associated with these processes, with XTH genes involved in all five categories. **b** Phylogenetic analysis of 31 *Sphagnum capillifolium* PME proteins based on the PME domain. **c** Phylogenetic analysis of 27 *Sphagnum capillifolium* XTH proteins, showing distinct clustering and functional specialization. **d** Phylogenetic analysis of 18 *Sphagnum capillifolium* Exo70 proteins, with five differentially expressed genes, three in Clade I and two in Clade III

Based on the GO enrichment analysis, this study conducted an in-depth examination of genes associated with the apoplast (GO:0048046), cell periphery (GO:0071944), cell wall (GO:0005618), extracellular region (GO:0005576), and external encapsulating structure (GO:0030312) processes (Fig. 4a). Analysis revealed that five genes from the *EXO* gene family are involved in the cell periphery, seven genes from the *PME* gene family are associated with the cell wall, external encapsulating structure, and cell periphery, and 13 genes from the *XTH* gene family are implicated in all five processes.

A dataset of 185 protein sequences, including 154 from *Arabidopsis thaliana* and 31 from *Sphagnum capillifolium*, was analyzed for phylogenetic relationships based on the PME domain (PF01095). The 31 ScPME proteins were classified into six major clades, with differentially expressed genes mainly in Clade I (Fig. 4b). Of the 31 proteins, 29 contained the Pectinesterase domain, and 14 had the pectin methylesterase inhibitor domain (PF04043), with Clade I genes featuring both domains (Fig. 4b). A third analysis of 70 sequences (43 from *A. thaliana* and 27 from *S. capillifolium*), using the Xyloglucan endo-transglycosylase C-terminus domain (PF06955) and Glycosyl hydrolases family 16 domain (PF00722), showed that the 27 ScXTH proteins clustered into a distinct clade, suggesting an evolutionary divergence and functional specialization of XTH proteins in *Sphagnum* (Fig. 4c). In a separate analysis of 45 sequences (27 from *A. thaliana* and 18 from *S. capillifolium*) based on the Exo70 exocyst complex C-terminal domain (PF03081), 18 ScEXO70 proteins formed three clades, with five differentially expressed genes—three in Clade I and two in Clade III (Fig. 4d).

### Structural and sequence comparison of *Sphagnum* genes with PME, XTH, and EXO70 A1 Homologs

**Fig. 5:**
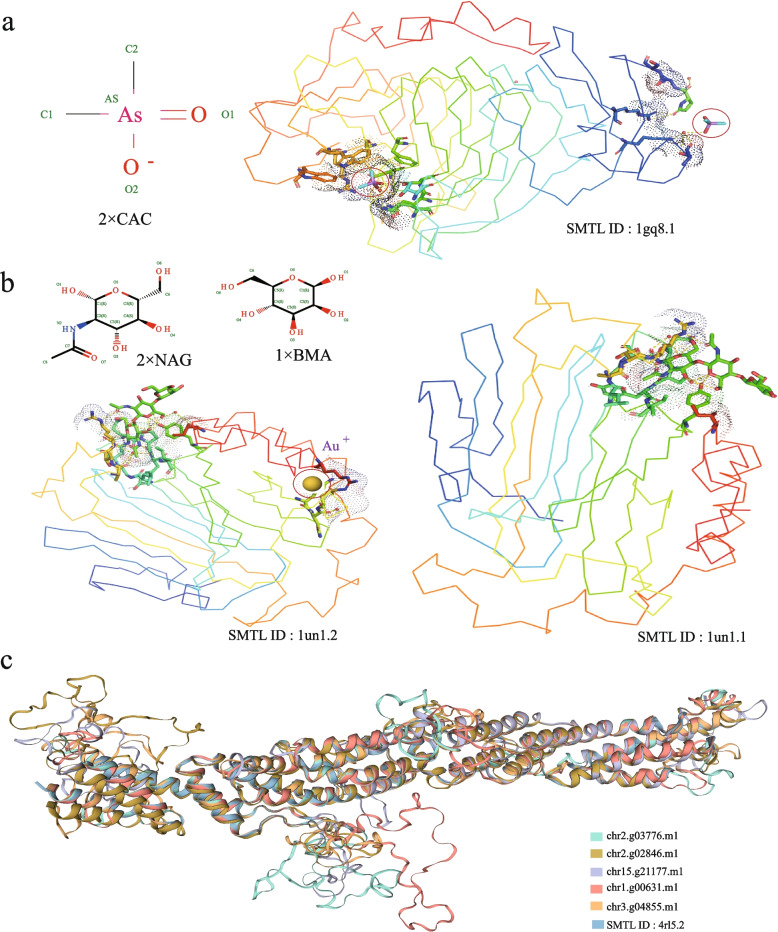
Structural and sequence comparison of *Sphagnum capillifolium* genes with PME, XTH, and EXO70 A1 homologs. **a** Structural comparison of *Sphagnum* PME genes with pectin methylesterase from *Carrot* (1 gq8.1). The *Sphagnum* PME homolog contains only one CAC ligand (left circle). **b** Structural of xyloglucan endotransglycosylase from *Populus tremula* × *tremuloides* (1un1.1 and 1un1.2). Significant differences in interactions and ligand compositions highlight the structural adaptations of *Sphagnum* XTH proteins. **c** Structural comparison of *Sphagnum* EXO70 A1 genes with *Arabidopsis* EXO70 A1 (4rl5.2), showing conserved β-sheet regions and variability in non-β-sheet regions, reflecting structural diversification in *Sphagnum* EXO70 proteins

The sequence identity between *Sphagnum* genes and pectin methylesterase (PME) from *Daucus carota* (1 gq8.1) ranges from 46.77% to 53.35%. The homology with the pectin methylesterase inhibitor from *Arabidopsis thaliana* (SMTL ID: 1x8z.1) is much lower, between 16.55% and 21.23% (Table S2). GMQE and QMEANDisCo Global scores suggest that PME alignments have higher model quality than those of invertase/pectin methylesterase inhibitors, indicating better structural conservation for PME (Table S2). In *D. carota* (1gq8.1), two CAC ligands stabilize the active site and modulate enzymatic activity (Fig. 5a). In contrast, the *Sphagnum* homolog contains only one CAC ligand, suggesting potential functional divergence.

Structural analysis of seven *Sphagnum* XTH proteins reveals close relationships with xyloglucan endotransglycosylase from *Populus tremula* x *tremuloides* (1un1.1), while five other XTH proteins align more closely with another *Populus* xyloglucan endotransglycosylase (1un1.2). Sequence identity with 1un1.1 ranges from 44.70% to 50.37%, with the highest homology found in chr13.g19208.m1 (Table S3). GMQE values range from 0.64 to 0.81, and QMEANDisCo scores between 0.51 and 0.83 indicate high structural reliability. In 1un1.1, ARG (188) is typically substituted by GLN and ASP in *Sphagnum*. Sequence identity with 1un1.2 is slightly higher, from 47.51% to 53.93%, with chr8.g13066.m1 showing the best alignment. Key differences in interactions include the substitution of ARG (188) with GLN and ASP in *Sphagnum*, and changes in ILE (255) and CYS (160) residues (Table S4). Both proteins contain two NAC and one BMA ligand, while 1un1.2 also includes an additional AU + metal complex, indicating a more complex structure (Fig. 5b).

The sequence identity between *Sphagnum* proteins and *Arabidopsis* EXO70 A1 (4rl5.2) ranges from 31.33% to 42.55%, with chr1.g00631.m1 showing the highest homology (42.55%) (Table S5). GMQE scores range from 0.44 to 0.52, and QMEANDisCo Global scores suggest acceptable model reliability, with values between 0.52 and 0.61. The *Sphagnum* EXO70 A1 homologs retain essential structural elements for function, but unlike PME and XTH proteins, 4rl5.2 lacks ligands. The *Sphagnum* EXO70 proteins exhibit greater structural variation, with conserved β-sheet regions (Fig. 5c) and more variability in non-β-sheet regions, indicating structural diversification among these proteins.

## Discussion

### Developmental adaptations and functional specialization of hyaline cells

The development of *Sphagnum* species, including *S. capillifolium*, demonstrates remarkable adaptations to the extreme challenges of wetland ecosystems, particularly persistent waterlogging and nutrient deficiency [28, 29]. The life cycle proceeds through a series of coordinated morphological transitions—from filamentous protonema and thalloid protonema to young and mature gametophytes [12]. Each stage represents a finely tuned developmental program optimized for peatland survival.

In the early stages, undifferentiated cells rich in chloroplasts and starch granules sustain high photosynthetic activity, establishing the energy base for subsequent cellular specialization [12]. As the gametophyte matures, hyaline cells gradually differentiate, marked by the initiation of secondary wall thickening, pore formation, and programmed cell death. These structural modifications compensate for the absence of vascular tissue, enabling efficient water storage and capillary transport— critical features in waterlogged habitats.

Our observations of *Sphagnum capillifolium* indicate that the initiation of hyaline cell differentiation occurs at the young gametophyte stage, differing from the pattern in which hyaline cells form at the apex of mature leaves [11, 12]. Interestingly, this differentiation may initiate simultaneously at multiple sites, not just from the leaf apex. This early developmental process includes a progressive reduction in chloroplast content, thickening of secondary walls, and eventual programmed cell death (PCD), resulting in mature hyaline cells equipped for water retention and movement.

Beyond water regulation, hyaline cells contribute significantly to nutrient acquisition and ecosystem functioning. Their hollow, porous architecture supports symbiotic associations with nitrogen-fixing bacteria and cyanobacteria, facilitating nutrient input in oligotrophic conditions [20, 30, 31]. Furthermore, hyaline cells act as hubs for microbial activity, promoting carbon cycling and contributing to peatland carbon sequestration [3, 17, 32]. Since the sporophyte cannot achieve axenic conditions, microbial symbiosis with *Sphagnum* persists during spore germination and culture. These specialized structures and their symbiotic relationships with microbes are thus essential in maintaining peatland hydrology, nutrient cycling, and ecosystem stability, highlighting their importance in wetland restoration and climate regulation.

### Transcriptomic insights into the regulatory patterns of hyaline cell formation

Transcriptomic analyses have revealed dynamic changes in gene expression accompanying hyaline cell differentiation. During the transition from young to mature gametophytes, numerous differentially expressed genes are enriched in Gene Ontology (GO) terms associated with cell wall metabolism and intercellular interactions. Notably, gene families such as *PME*, *XTH*, and *EXO70* are prominently involved, playing roles in cell wall remodeling, cellular differentiation, and extracellular organization [25, 26, 33–38]. Among them, pectin methylesterases (PMEs) are of particular importance. These enzymes mediate the demethylesterification of homogalacturonans, thereby regulating cell wall remodeling [39, 40]. Other pectin-related enzymes—including pectatelyases, pectinase, and PMEI proteins—work in concert to modulate wall loosening and restructuring [41–43]. While PME proteins in *Sphagnum* share high sequence similarity with homologs in *Arabidopsis thaliana*, notable differences in their active sites and ligand affinities suggest adaptive modifications tuned to the demands of wetland environments.

The *XTH* gene family, which encodes xyloglucan endotransglycosylase/hydrolases, is involved in cell wall expansion through xyloglucan remodeling [44–46]. In *Sphagnum*, structural analyses of XTH proteins uncovered potential functional divergence. For example, in the structural variant 1un1.1, the conserved residue ARG (188) is frequently replaced by GLN or ASP in *Sphagnum*, possibly altering substrate specificity or enzymatic activity [47]. By contrast, comparison with variant 1un1.2 revealed slightly higher sequence identity, suggesting closer evolutionary relatedness or conservation of function. These structural variants also differ in ligand composition; while both contain two NAC and one BMA ligands, 1un1.2 uniquely possesses an AU + metal complex, suggesting added functional complexity. Such substitutions highlight the dynamic evolution of XTH proteins and their adaptive significance in supporting the specialized physiology of hyaline cells.

The *EXO70* gene family, encoding a component of the exocyst complex, plays a central role in vesicle targeting, membrane fusion, and extracellular secretion [37]. In *Sphagnum*, EXO70 proteins exhibit high conservation in the β-sheet domains but considerable variation in the non-β-sheet regions compared to *Arabidopsis*. These differences may reflect specific adaptations to the demands of hyaline cell development, particularly the extensive secretion processes required for wall thickening and pore formation.

### Conclusion and future directions

Through the integration of detailed morphological observations and transcriptomic profiling across multiple gametophyte developmental stages, this study provides novel insights into the developmental regulation of hyaline cell formation in *Sphagnum*. Differential expression of several gene families—particularly those associated with cell wall modification and cellular differentiation, including *PME*, *XTH*, and *EXO70*—highlights their potential roles in orchestrating the formation of specialized cell types. These structures are critical for the ecological functions of *Sphagnum*, notably in facilitating water retention, promoting nutrient cycling, and enhancing carbon sequestration within peatland ecosystems.

Future research should further explore the functional roles of these candidate genes under environmental stressors such as drought, temperature fluctuations, and nutrient limitations. The preliminary establishment of a transient transformation system in *Sphagnum* provides a valuable foundation for gene function validation, expanding its potential as a model organism for molecular studies and enabling deeper investigation into the genetic basis of its unique traits [48]. Combining functional studies with comparative analyses across diverse *Sphagnum* species will help elucidate the genetic mechanisms of ecological adaptation. Furthermore, a comprehensive understanding of hyaline cell development will support applied research in peatland restoration, climate change mitigation, and the sustainable utilization of moss resources. Continued investigation linking *Sphagnum* biology with ecosystem function is essential in the face of ongoing environmental change.

## Materials and methods

### Plant material and morphological observations

The spores of *Sphagnum capillifolium*, collected from Hani (Jilin Province), were sterilized using a 1% sodium hypochlorite solution and washed five times with sterile water. To prepare the spore suspension, the spores were rehydrated in sterile water and gently agitated. Following the protocol previously established for *Sphagnum* cultivation [49, 50], 200 μl of the spore suspension was evenly spread onto Knop's solid medium in Petri dishes, which were sealed and incubated in the dark for 3 days. After this, the dishes were transferred to a controlled environment at 24 ± 2 °C, with a 16-h light/8-h dark photoperiod and a light intensity of 100 μmol m⁻^2^ s⁻^1^. Approximately 7 days later, bulges appeared on the adaxial surface of the spores, signaling the formation of germinating spores that gradually developed into filamentous protonema. Under the same growth conditions, the filamentous protonema transformed into thalloid protonema after about 5 days. As the thalloid protonema began to form gametophytes-like structures, young gametophytes gradually emerged, a process that took approximately one week. The young gametophytes were then transferred to fresh Knop's solid medium and cultivated at 25 °C under a 12-h light/12-h dark photoperiod.

At designated time points, samples of filamentous protonema (7 days), thalloid protonema (12 days), young gametophytes (17 days), and mature gametophytes (2 months) were collected for morphological observation and transcriptomic analysis. Fresh samples at each stage were carefully harvested and photographed under a stereomicroscope (Nikon Corporation, Tokyo, Japan). Detailed documentation of the developmental transitions—from spore germination to protonemal formation and subsequent gametophyte development—was conducted to track cellular differentiation at each stage. Since the sporophyte cannot achieve axenic conditions, microbial contamination remained present during spore germination and culture. The identification of *Sphagnum capillifolium* was formally carried out by Xian-Lin Guo (Chengdu Institute of Biology, Chinese Academy of Sciences), and voucher specimens have been deposited in the herbarium of the Chengdu Institute of Biology, Chinese Academy of Sciences (CDBI).

### RNA Extraction and Sequencing

Transcriptome samples were collected from four developmental stages, with three biological replicates for each stage, totaling 12 samples (Table S1). RNA extraction, quality assessment, library construction, and sequencing followed standard protocols. First, the integrity of RNA was assessed using 1% agarose gel electrophoresis to ensure no degradation or contamination. RNA concentration and purity were then measured using a NanoPhotometer® spectrophotometer, and RNA integrity was further confirmed using the RNA Nano 6000 Assay Kit on the Bioanalyzer 2100 system. Total RNA was used for library construction, with mRNA isolated using Oligo (dT)-coated capture beads, purified with binding and washing buffers. mRNA was then randomly fragmented into 100–200 nt fragments in fragmentation buffer and reverse-transcribed into cDNA. The synthesized cDNA was purified using DNA clean beads, followed by adapter ligation and a one-step PCR reaction with pre-mixed adapters containing unique molecular identifier regions (UMIs). PCR products were purified again using DNA clean beads and recovered with nuclease-free H_2_O. After library preparation, samples from different stages were pooled and sequenced on the Illumina NovaSeq 6000 platform, generating 6 Gb of raw data with 150 nt paired-end reads. The raw data of RNA sequences were submitted to the NGDC (National Genomics Data Center: https://ngdc.cncb.ac.cn/) under the BioProject number PRJCA017193.

### Differential gene expression

UMI sequences on each read were identified by UMI-tools v1.0.0 [51], and reads with UMIs were used for the subsequent analysis. To identify the duplicated reads, UMIs were initially removed from the UMI reads, and the remaining parts of each read were mapped to the reference genome using Hisat2 v 2.1.0. [52]. Reads that mapped to the same location on the *Sphagnum capillifolium* genome (NGDC: PRJCA017193) were identified as duplicated reads. Then, the UMIs on each read were recalled, and the duplicated reads with the same UMI were identified as non-natural duplications, which were subsequently removed from the processed data. HTSeq v0.6.1 [53] was used to count the read numbers mapped to each gene. Then, the FPKM of each gene was calculated based on the length of the gene, and the read count was mapped to the gene. Differential gene expression across developmental stages (three biological replicates per condition) was analyzed using the DESeq R package v1.18.0 [54]. P-values were adjusted for multiple testing using the Benjamini-Hochberg method [55], and genes with an adjusted P-value < 0.05 and |log2(FoldChange)| > 1 to identify differentially expressed genes (DEGs) were considered differentially expressed.

### GO enrichment and KEGG pathway analysis of DEGs

GO enrichment analysis of differentially expressed genes was performed using the GOseq R package, correcting for gene length bias [56]. GO terms with a corrected P-value < 0.05 were considered significantly enriched. Differentially expressed genes were further subjected to GO enrichment and KEGG pathway enrichment analyses. GO analysis (http://geneontology.org/) was performed using a hypergeometric test, and REVIGO software (http://revigo.irb.hr/) was used to visualize GO cellular component terms significantly associated with the cell wall after redundant terms were removed. KEGG enrichment analysis (http://www.genome.jp/kegg) was conducted by comparing genes involved in metabolic or signal transduction pathways with the whole genome background. The significantly induced or repressed KEGG metabolic pathways were identified by the KEGG enrichment analysis using the TBtools v 1.046 [57].

### Phylogenetic analysis

The genome sequences of *Arabidopsis thaliana* (NCBI: GCA_000001735.2) and *Sphagnum capillifolium* (NGDC: PRJCA017193) were retrieved from the National Center for Biotechnology Information (NCBI) genome database (https://www.ncbi.nlm.nih.gov/) and the National Genomics Data Center (NGDC) genome database (https://ngdc.cncb.ac.cn/), respectively. To identify the relevant protein families, the PME (PF01095), XTH (PF00012), and EXO70 (PF000113) Pfam models were downloaded from the Pfam database (http://pfam.xfam.org/). HMMER v3.2.1 software [58] was used to extract candidate *PME*, *XTH*, and *EXO70* genes from genome sequences of *Arabidopsis thaliana* and *Sphagnum capillifolium* based on the Pfam domain models. The identified gene sequences were validated using the NCBI Conserved Domain Database (CDD) [59] and the Simple Modular Architecture Research Tool (SMART) database (http://smart.embl-heidelberg.de/) [60]. This step was carried out to confirm the presence of functional domains and ensure that the sequences were accurately identified for subsequent analysis.

Three protein datasets were analyzed to explore phylogenetic relationships. For the PME domain, 185 protein sequences were examined, including 154 from *A. thaliana* and 31 from *S. capillifolium*. For the Exo70 domain, 45 sequences were analyzed, consisting of 27 from *Arabidopsis thaliana* and 18 from *S. capillifolium*. Additionally, 70 sequences related to the XTH domain were evaluated, comprising 43 from *Arabidopsis thaliana* and 27 from *S. capillifolium*. Validated sequences for PME, XTH, and Exo70 proteins from both species were imported into Geneious Prime (version 2021) and aligned using the Geneious Alignment method [61]. Multiple sequence alignment was performed using MUSCLE v3.8.1551 with default parameters [62]. The aligned sequences were used to construct phylogenetic trees using the PhyloSuite v.1.2.2 [63], applying the Jones-Taylor-Thornton (JTT) model for amino acid substitution with a bootstrap value of 1000. The resulting trees were visualized using iTOL v7 [64] to illustrate the evolutionary relationships between the identified proteins.

### 3D structure prediction of PME, XTH, and EXO70 Proteins

The 3D structures of PME, XTH, and EXO70 proteins were predicted using SWISS-MODEL (https://swissmodel.expasy.org/) [65]. All of the figures were prepared using PyMol 2.5 (The PyMOL Molecular Graphics System, Schrödinger, LLC). and all interactions were detected using the PLIP algorithm [66]. Ligand-binding sites were identified using SWISS-MODEL to investigate potential functional interactions of the proteins. These structural models provide insights into the potential mechanisms underlying protein function and interactions.

## Supplementary Information

Supplementary Material 1.

## Data Availability

The *Sphagnum capillifolium* genome project and transcriptome sequencing data has been deposited at the NGDC(National Genomics Data Center: https://ngdc.cncb.ac.cn/) under the BioProject number PRJCA017193.

## Abbreviations

ARG: Arginine
ASP: Aspartic acid
ASN: Asparagine
AU +: Gold ion
CDBI: Herbarium of chengdu institute of biology, Chinese Academy of Sciences
CDD: Conserved domain database
CYS: Cysteine
cDNA: Complementary DNA
DEGs: Differentially expressed genes
EXO70 A1: Exocyst subunit 70A1
FP: Filamentous protonema stage
Gb: Gigabyte
GLN: Glutamine
GMQE: Global model quality estimation
GO: Gene ontology
HIS: Histidine
ILE: Isoleucine
JTT: Jones-taylor-thornton model for amino acid substitution
KEGG: Kyoto encyclopedia of genes and genomes
NCBI: National center for biotechnology information
NGDC: National genomics data center
Nt: Nucleotide
PCA: Principal component analysis
PDB: Protein data bank
PLIP: Protein-ligand interaction profiler
PME: Pectin methylesterase (PF01095, Pfam model)
QMEANDisCo: Qualitative model energy analysis distance constraints
RNA: Ribonucleic acid
TP: Thalloid protonema stage
UMI: Unique molecular identifier
XTH: Xyloglucan endotransglucosylase/Hydrolase (PF00012, Pfam model)
YG: Young gametophyte stage
